# Impaired empathy but no theory of mind deficits in adult attention deficit hyperactivity disorder

**DOI:** 10.1002/brb3.1401

**Published:** 2019-09-02

**Authors:** Mona Abdel‐Hamid, Franziska Niklewski, Philipp Heßmann, Nika Guberina, Melanie Kownatka, Markus Kraemer, Norbert Scherbaum, Isabel Dziobek, Claudia Bartels, Jens Wiltfang, Bernhard Kis

**Affiliations:** ^1^ Department of Psychiatry and Psychotherapy University Medical Center Göttingen (UMG) Göttingen Germany; ^2^ LVR Hospital Essen Department of Psychiatry and Psychotherapy Faculty of Medicine University of Duisburg‐Essen Essen Germany; ^3^ Institute of Diagnostic and Interventional Radiology and Neuroradiology University Hospital Essen Essen Germany; ^4^ Department of Neurology Alfried Krupp von Bohlen and Halbach Hospital Essen Germany; ^5^ Department of Neurology Heinrich Heine University Düsseldorf Düsseldorf Germany; ^6^ Berlin School of Mind and Brain Humboldt University Berlin Germany; ^7^ Department of Psychiatry, Psychotherapy and Psychosomatics St. Elisabeth-Hospital Niederwenigern, Contilia Group Hattingen Germany

**Keywords:** adult ADHD, comorbidity, empathy, executive functions, theory of mind

## Abstract

**Objective:**

The cognitive capacity to change perspective is referred to as theory of mind (ToM). ToM deficits are detectable in a variety of psychiatric and neurological disorders. Since executive abilities are closely associated with ToM skills, we suspected that due to a common neuropsychological basis, ToM deficits exist in treatment‐naïve adults with attention‐deficit/hyperactivity disorder (ADHD).

**Methods:**

Performance of treatment‐naïve adults with ADHD (*N* = 30) in tasks assessing executive functions (Trail Making Test, Stroop color–word test, and Test Battery for Attentional Performance), empathy skills (Cambridge Behaviour Scale), and ToM (Movie for Assessment of Social Cognition) was compared with that of a healthy control group (*N* = 30) matched according to basic demographic variables.

**Results:**

Compared to healthy controls, treatment‐naïve adults with ADHD showed deficits in various executive functions and the ability to empathize (all *p* < .05). However, no performance differences were found with regard to ToM (all n.s.).

**Conclusions:**

Since studies in juveniles with ADHD often show impaired ToM performance, it is conceivable that ToM deficits may become attenuated due to neuronal development in adolescence. Furthermore, our findings imply that ToM impairments, even when present in adult ADHD, appear to be independent of executive deficits and might be explained by comorbid disorders.

## Graphical abstract

This study explored the extent to which social interaction problems of treatment‐naïve adults with ADHD can be attributed to deficits in theory of mind using established neuropsychological tests. The results revealed that adults with ADHD showed impairment in various executive functions and the ability to empathize but no theory of mind deficits. Our findings imply that ToM impairments, even when present in adult ADHD, appear to be independent of executive deficits and might be explained by comorbid disorders.

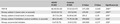

## SIGNIFICANT OUTCOMES

- The lack of empathy skills in treatment‐naïve adults with ADHD is a robust result.
- Contrary to previous results, unmedicated adult ADHD patients showed no ToM deficits.

## LIMITATIONS

- We did not assess levels of social functioning. Future studies should therefore assess the extent to which there is any impairment in downstream functions and to what extent these issues are related to ToM or empathy deficits.
- This exploratory study with relatively small sample sizes provides the first evidence of differential associations between cognitive and emotional perspective taking with executive functions but requires further replication in larger study samples.

## 1 INTRODUCTION

The term social cognition describes the general ability to visually and auditorily perceive, interpret, and behave based on important stimuli in a social context (Wiener & Rybakowski, 2006). Such social cognition is an umbrella term covering various skills. The cognitive capacity to change perspective to take somebody else's point of view is referred to as theory of mind (ToM; Premack & Woodruff, 1978). As a result of this cognitive perspective taking, the thoughts and actions of others can be interpreted, comprehended, and predicted. In addition to ToM, the term social cognition also comprises empathy and the recognition and interpretation of emotions in facial expressions, prosody and parlance, and gestures and postures, as well as humor processing and decision making in social contexts. All these skills are crucial for successful social interaction (Uekermann et al., 2010). A clear distinction between ToM and empathy is hampered by an increasing differentiation of these concepts into cognitive and affective elements. On the one hand, we follow the definition that empathy involves taking over of and responding to the emotional inner perspective of an observed person, regardless of the intentions of the person in focus (Förstl & Förstl, 2012). On the other hand, ToM is a cognitive achievement in actively empathizing with another person's state of mind and emotions (Premack & Woodruff, 1978). While empathy develops in human ontogenesis before complex metacognitive skills such as ToM, it can also be shown by primates and other mammals. ToM is attributed to anthropoids and humans (Schievenhöfel & Förstl, 2012).

Current evidence suggests that ToM deficits are detectable in a variety of psychiatric and neurological disorders, including attention‐deficit/hyperactivity disorder (ADHD; Uekermann et al., 2010) autism, (Hoogenhout & Malcolm‐Smith, 2017) schizophrenia spectrum disorders, (Fernandes, Cajao, Lopes, Jeronimo, & Barahona‐Correa, 2018) frontal lobe and right hemisphere lesions, (Baldo, Kacinik, Moncrief, Beghin, & Dronkers, 2016) personality disorders, (Bilotta et al., 2018; Németh et al., 2018) multiple sclerosis, (Isernia et al., 2019) and dementias, (Moreau, Rauzy, Viallet, & Champagne‐Lavau, 2016) and thus detrimentally affect everyday social life. In the context of these disorders, there is ongoing discussion as to whether ToM abilities are a specific, independent capability, or whether they represent a nonspecific skill, that is, based on a combination of different abilities such as executive functions or memory skills (Mary et al., 2016; Sommer, Döhnel, Schuwerk, & Hajak, 2012).

Executive function is a collective term for different control processes that are responsible for the coordination, configuration, monitoring, and evaluation of sensory, cognitive, and motor systems (Goschke, 2002). They serve to self‐regulate purposeful behaviors by suppressing inadequate, impulsive responses and enabling the planning of new actions (Luria, 1980; Milner, Petrides, & Smith, 1984). Furthermore, executive functions allow adaptation to new situations by changing cognitive settings and problem‐solving by selecting and holding task‐relevant information in mind (Shallice, 1988). In addition, executive functions include abstract reasoning, hypothesis generation, temporal sequencing, and prospective memory (Giancola & Moss, 1998; Smith & Jonides, 1999). Deficits in executive functions, however, are particularly and strongly associated with ADHD (Tarver, Daley, & Sayal, 2015; Uekermann et al., 2010).

The link of both constructs, executive functions and ToM, in ADHD has been explicitly addressed in a small number of studies. Mary et al. (2016) clearly demonstrated in children with ADHD that ToM deficits were due to impaired executive functions. Gonzalez‐Gadea et al. (2013) pointed out, however, that executive performance and ToM skills can vary greatly among adults with ADHD.

Screening and lesion studies in these patient groups have also helped to identify underlying neuronal networks ascribed to ToM and executive functions. Wade et al. (2018) summarized that the different ToM abilities are related to areas such as the bilateral temporoparietal junction (the inferior parietal lobule at the junction with the posterior temporal cortex), medial prefrontal cortex (including the anterior paracingulate cortex), precuneus/posterior cingulate cortex (including anterior paracingulate cortex), and sulcus temporalis superior/middle temporal gyrus. Overall, ToM skills have been attributed to a widespread fronto‐temporo‐parietal functional network. Different executive functions are based on simultaneous activity in areas such as the dorsolateral prefrontal cortex, ventromedial prefrontal cortex, ventrolateral prefrontal cortex, inferior parietal lobule, anterior cingulate cortex, and superior parietal lobule (Wade et al., 2018). The authors concluded that ToM skills and executive capabilities are based on partially overlapping networks comprising the medial prefrontal cortex, inferior parietal lobule, temporoparietal junction, and inferior frontal gyrus. These areas seem to provide a common neuronal basis for both functional outcomes, ToM, and executive functions.

In light of the multifaceted association of ToM with executive functions, the impact of ToM on ADHD has to be further elucidated. Adult patients with ADHD often seek help from clinicians since the consequences of ADHD symptoms cause high levels of distress. ADHD is considered a heterogeneous neurodevelopmental disorder diagnosed in childhood that persists into adulthood. ADHD is generally associated with disorders of attention, concentration, and organization as well as hyperactivity, impulsivity, and emotional lability. A distinction is made between three subtypes: inattentive subtype, hyperactive–impulsive subtype, and mixed subtype. Affected clients often report difficulties in social relationships (i.e., in the family, in the workplace) due to inattentive, impulsive, or generally socially inadequate behaviour (Nijmeijer et al., 2008). There is a series of studies in children with ADHD that showed that ADHD is often accompanied by impairments in social cognition (Pineda‐Alhucema, Aristizabal, Escudero‐Cabarcas, Acosta‐Lopez, & Velez, 2018). However, due to contradictory results, it remains unclear whether ToM deficits persist into adulthood (Bora & Pantelis, 2016). Recent studies investigating adult patients with ADHD and their ability to perceive prosody and emotional faces showed that they displayed impaired performance, especially in the perception of angry emotional statements. These results were independent of executive skills (Bisch et al., 2016; Kis et al., 2017). Nevertheless, studies investigating social cognition in adults with ADHD (Uekermann et al., 2010), especially the association between social cognition and executive functions in adults with ADHD, are still rare.

### 1.1 Aims of the study

We suspect that due to a common neuronal basis for ToM abilities and executive functions, ToM deficits exist in treatment‐naïve adults with ADHD. However, the extent to which these social cognitive impairments are selective, that is, constitute independent deficits or are connected to the executive dysfunction that is common in ADHD, needs to be clarified. This could be of great importance for the selection or development of specific treatment approaches. To reduce the negative consequences and the associated suffering that is experienced in adults with ADHD, either executive abilities or social cognitive skills would have to be specifically trained.

Furthermore, we aim to explore whether various aspects of social cognition, that is, ToM and empathy, are equally impaired or whether they are differentially affected.

## 2 MATERIALS AND METHODS

### 2.1 Study sample

The study was approved by the Ethics Committee of the University of Duisburg‐Essen, Germany, and all participants provided informed consent according to the Declaration of Helsinki. Patients were contacted for study participation at the ADHD outpatient clinic of the Department of Psychiatry and Psychotherapy, University of Duisburg‐Essen. Participants in the control group were recruited by announcements in public places. Recruitment took place from 2012 to 2017.

Inclusion criteria for the patient group were an initial diagnosis of adult ADHD (DSM‐IV‐TR; Saß, Wittchen, & Zaudig, 2003) and age ≥18 years. All participants had to be treatment‐naïve with regard to ADHD, that is, without past or current psychiatric, psychological, or medical treatment. We used WURS‐k (Retz‐Junginger et al., 2002) and ADHS‐SB (Rosler et al., 2004) to detect attention difficulties, disorganization, hyperactivity, and impulsivity. For eligibility, neurological and psychiatric comorbidities had to be excluded, particularly with regard to affective disorders as well as alcohol and substance abuse (except for smoking/nicotine). All participants were fluent in German, and due to inclusion and exclusion criteria, neurological disorders and thus at least cerebro‐organic language or speech disorders were excluded.

### 2.2 IQ estimation

Patient and control groups underwent a standardized neuropsychological test battery, including tests for IQ estimation and executive functions. For measuring practical intelligence, we used the “Similarities” and “Picture Completion” subtests from the reduced Wechsler Intelligence Test (WIP; Dahl, 1972). The Multiple‐Choice Vocabulary Intelligence Test (MWT‐B; Lehrl, 1995) was used to estimate verbal IQ.

### 2.3 Executive functions

To investigate executive functions, standard neuropsychological measures were used that capture essential aspects of executive functions, that is, visual scanning, inhibition, attentional performance, and attention shifting. Therefore, the Trail Making Test, subtests A and B (TMT‐A, TMT‐B; Reitan, 1992), was used to assess visual scanning and attention shifting/cognitive flexibility as well as working memory skills (Sánchez‐cubillo et al., 2009). Both the Stroop color–word test (Stroop, 1935) and the “Go/No‐Go” test from the computer‐based Test Battery for Attentional Performance (TAP, Zimmermann & Fimm, 2002) required inhibition of dominant reaction tendencies and attentional performance.

### 2.4 Social cognition

To investigate social cognition, psychometric instruments were chosen to cover emotional (empathy) and cognitive perspective taking (ToM). Thus, adult empathy was assessed by the Cambridge Behaviour Scale (Baron‐Cohen & Wheelwright, 2004). This is a self‐assessment tool that consists of 60 items, 40 of which are designed for the quick and easy detection of empathy. The remaining 20 items are distractors. The sum score served as the outcome parameter (Kis et al., 2017).

The Movie for Assessment of Social Cognition (MASC) is a video‐based psychometric instrument for the measurement of mental state attribution skills, particularly ToM (Dziobek et al., 2006). It consists of a 15‐min movie portraying two men and two women spending an evening together. The movie focuses on the social interactions between these characters. In this scenario, different protagonists react with joy, fear, anger, appreciation, jealousy, embarrassment, or reluctance in social interactions. The movie stops at certain intervals and asks the spectator to answer questions referring to the previous scene. These questions demand that the spectator put himself or herself into the depicted scene and require ToM skills since the viewer has to comprehend the protagonist's thoughts, emotions, or intentions. There are four answer options, allowing a differentiation between “no ToM,” “less ToM,” “normal ToM,” and “exceeding ToM.” In detail, this means that only one of the four answer options is correct (“normal ToM”). An overinterpretation of a protagonist's thoughts, emotions, or intentions leads to a rating as “exceeding ToM.” An overly literal interpretation of the situation results in a classification of “less ToM.” A purely factual evaluation without consideration of the perspective of the portrayed protagonist leads to the classification “no ToM.” The information used by the MASC test participants is based on different sources. Alongside facial expressions, verbal utterances and behavior of the protagonists are required as a source of information. Occasional control questions are asked to control for memory and attentional performance.

### 2.5 Statistics

Frequencies were used as descriptive statistics for categorical data. Mean and standard deviations were calculated for continuous data. Intergroup comparisons were carried out using independent Student's *t* tests. When the data were not normally distributed (Kolmogorov–Smirnov test for normality of continuous data and Levene's test for variance homogeneity), the nonparametric Mann–Whitney *U* test was performed. All statistical analyses were two‐sided with the significance threshold set at .05 and were conducted with SPSS Statistics, version 24.0.

## 3 RESULTS

### 3.1 Study sample

The patient group included 30 adult treatment‐naïve patients with a confirmed ADHD diagnosis based on the DSM‐IV‐TR (Saß et al., 2003). Fifteen patients were male, and 15 were female. There were 13 patients with the inattentive subtype, 16 with the combined subtype, and 1 with the hyperactive subtype. Ages ranged between 23 and 49 years (*M* = 34.50 years; *SD* = 6.81). The average verbal intelligence reached 108.91 (*SD* = 7.67) and 112.10 (*SD* = 7.86) for practical intelligence. The average school education was 11.10 years (*SD* = 1.56), and the average amount of higher education was 3.30 years (*SD* = 1.55). Nine of the 30 patients stated that they smoked.

The ADHD patient group and the control group were matched by demographic variables such as age, sex, IQ, and duration and level of education in order to control for the influence of those variables. The control group (*N* = 30) consisted of 15 males and 15 females. They were between 18 and 57 years old (*M* = 35.83 years, *SD* = 11.68). Verbal intelligence averaged 110.22 (*SD* = 8.72), and practical intelligence averaged 108.87 (*SD* = 9.03). The control group had an average amount of school education of 12.07 years (*SD* = 1.55) and 3.45 years (*SD* = 2.26) of higher education. There were five smokers in the control group.

### 3.2 Executive functions

In terms of executive function performance, patients with ADHD displayed a typical performance profile. They performed significantly worse in the vast majority of the tests for executive functions than the healthy control subjects (Table 1).

**Table 1:** Set‐shifting and inhibition in the ADHD and control groups

	ADHD *M* (*SD*)	Controls *M* (*SD*)	*U*‐Value	*Z*‐Value	Significance (*p*)
TMT‐B	86.40 (55.42)	57.13 (13.57)	271.500	−2.640	.008**
Stroop, subtest 1 (name color, in seconds)	53.03 (14.71)	45.9 (6.94)	274.00	−2.607	.009**
Stroop, subtest 2 (read word, in seconds)	35.7 (11.24)	31.00 (6.24)	291.5	−2.35	.019*
Stroop, subtest 3 (inhibition control condition, in seconds)	88.6 (25.80)	75.70 (14.28)	290.000	−2.267	.018*
Stroop, uncorrected mistakes	0.67 (0.71)	0.43 (1.01)	324.000	−2.177	.030*

Note

Level of significance: *p < .05; **p < .01; ***p < .001.

Abbreviations: ADHD, attention‐deficit/hyperactivity disorder; *M*, mean; *SD*, standard deviation; TMT‐B, Trail Making Test, part B.

In detail, a nonsignificant difference occurred when comparing the performance on the TMT‐A between the two groups (*t* (58) = 0.617, *p* = .54), implying similar visual scanning abilities in both groups. The TMT‐B, a test that requires attention shifting/cognitive flexibility as well as working memory skills (Sánchez‐cubillo et al., 2009), yielded a significant difference in processing time in favor of the healthy control group (Table 1). Significant differences were also found in all three subtests of the Stroop color–word test, especially in subtest 3 for processing time with more uncorrected errors (Table 1), indicative of poorer inhibition control in the ADHD group. Regarding the response time in the Go/No‐Go test from the TAP, we did not find a significantly faster response time in the patient group than in the control group (*t* (58) = −0.544, *p* = .588), but patients committed more mistakes (*t* (58) = −2.08, *p* = .042*).

### 3.3 Social cognition

With respect to our central hypotheses/central constructs, the ADHD group had a significantly lower average score on the Cambridge Behaviour Scale than the control group (Mann–Whitney *U* = 311.000, *Z* = −2.058, *p* = .04*), thus showing impaired empathy (Table 2).

**Table 2:** Empathy and theory of mind skills in the ADHD and control groups

	ADHD *M* (*SD*)	Controls *M* (*SD*)	*t*‐Value	*df*	*U*‐Value	*Z*‐Value	Significance (*p*)
Empathy	35.47 (14.35)	42.87 (11.23)			311.000	−2.058	.04*
No ToM	2.67 (1.90)	1.77 (1.38)			329.000	−1.823	.068
Less ToM	4.17 (2.74)	4.07 (2.36)			444.500	−0.082	.934
Normal ToM	32.20 (5.18)	34.43 (4.25)	−1.825	58			.073
Exceeding ToM	5.97 (3.78)	4.53 (2.42)			348.00	−1.523	.128

Note

Level of significance: *p < .05; **p < .01; ***p < .001.

Abbreviations: ADHD, attention‐deficit/hyperactivity disorder; *df*, degrees of freedom; *M*, mean; *SD*, standard deviation; ToM, theory of mind.

In contrast, there were no significant differences between adult participants with ADHD and healthy controls in terms of ToM performance in the MASC, although patients performed slightly poorer in particular MASC tasks than controls (Table 2). However, the finding of significantly more attention errors in the ADHD group highlights the presence of ADHD symptoms (Mann–Whitney *U* = 311.000, *Z* = −2.058, *p* = .021*).

### 3.4 Correlation and multiple regression analysis

We calculated Spearman's Rho between ToM, empathy, and the various variables of executive functioning separately for ADHD patients and healthy controls. Within the healthy control group, we could not show any correlations between ToM, empathy, and executive functions. Correlations between empathy and executive measures for the ADHD group are displayed in Table 3.

**Table 3:** Correlations between empathy/ToM and executive measures for the ADHD group

		TMT‐A	TMT‐B	Executive control questions (MASC)
Empathy	*r* *p*	−.508 .004**	−.497 .005**	−.361 .050*
Theory of Mind (“normal ToM,” MASC)	*r* *p*		−.417 .022*	

Note

*r* = correlation coefficient (Spearman's correlation). Level of significance: *p < .05; **p < .01; ***p < .001

Afterward, we conducted moderation analyzes, a form of multiple regression analysis. We defined empathy and ToM as dependent variables and group membership as moderating variable. We set TMT‐A, TMT‐B, and executive control questions as independent variables/predictors. We planned to investigate how group affiliation influenced the relationship between dependent and independent variables. Regression models were calculated in which a significant interaction demonstrates a significant influence of the moderating variable. Group affiliation showed significant influences on the relation between empathy and TMT‐A (*t* = −2.6045, *p* = .0117), empathy, and TMT‐B (*t* = −2.6608, *p* = .0102) as well as an almost significant moderating effect on the relation between empathy and executive control questions (*t* = −1.9827, *p* = .0532). When analyzing ToM, we were not able to prove that cognitive flexibility as measured by the TMT‐B had a different influence on ToM in both groups (*t* = −0.233, *p* = .9815).

## 4 DISCUSSION

In general, the pattern of executive performance confirms those impairments that are concordant with the diagnostically and scientifically acknowledged ADHD symptomatology (American Psychiatric Association, 2013; World Health Organization, 2010). We were able to demonstrate typical deficits, such as impaired attention shifting and lower working memory skills. Poor impulse control was also demonstrated by the impaired ability to suppress dominant reaction tendencies as measured by committed mistakes. Taken together, we were able to broadly replicate executive dysfunction in our sample of adult, treatment‐naïve patients with ADHD.

Likewise, our results are in line with the robust finding of a lack of empathy skills in adults with ADHD. Additionally, Kis et al. (2017) were able to demonstrate an impaired perception of prosody as well as an impaired affective change of perspective. Groen, Heijer, Fuermaier, Althaus, and Tucha, (2018) also assumed a connection between traits of ADHD and emotional aspects of empathy. Furthermore, our results imply that empathy and ToM represent two independent skills of social cognition since empathy did not correlate with ToM at all, neither in the healthy control nor in the ADHD group. The Cambridge Behaviour Scale was initially developed to measure empathy unidimensionally. Baron‐Cohen and Wheelwright (2004) followed the argument that cognitive and affective aspects of an empathic response cannot be separated thoroughly. However, empathy measures based on self‐report in contrast to performance‐based measures seem not to be as closely associated as predicted suggesting different underlying constructs (Murphy & Lilienfeld, 2019). Here, even our results imply that empathy skills seem to be related to executive capabilities. On the one hand, it remains open to debate whether this test captures empathy as defined or if it additionally relies on cognitive performance. Due to our results showing a close relation between empathy and cognitive functions, it would also be conceivable that empathy is a complex, multifaceted ability. However, it cannot be excluded that performance‐based empathy tests would have revealed different results. In terms of empathy and ToM, further basic and theoretical research is therefore required in order to ensure a clear definition and differentiation between these two concepts.

Regarding the cognitive perspective taking, that is, ToM, we yielded novel and divergent results. A major finding of the present study was that treatment‐naïve, adult ADHD patients showed no outstanding ToM deficits. On the one hand, our findings can be interpreted with regard to Bora and Pantelis (2016) who suggested that social cognitive deficits may improve due to neuronal development and maturation or increasing social experience in adolescence. However, according to our findings, this maturation may only relate to the acquisition of cognitive changes in perspective, while empathy skills may represent a noncompensable deficit. On the other hand, it appears that ToM deficits, when they are present in adults with ADHD, seem to be independent of executive functions and represent an impairment of their own. Since we were able to prove that cognitive flexibility did not have a different influence on ToM abilities in both groups, this result could show that among those adults with ADHD portrayed in this study, ToM skills were independent of impairments in executive functioning. The assumption of Bora and Pantelis (2016) stating that the neurodevelopmental disorder ADHD and its associated cognitive deficits can be very heterogeneous among those persons affected is supported by our study. Despite a partially overlapping neuronal network responsible for ToM and executive skills, the neuronal differences might account for our results. Thus, our findings seem to contradict the assumption of Wade et al. (2018), who assumed a basic contribution from executive functions to ToM skills.

It is conceivable that if adults with ADHD show impaired ToM performance, this deficit could be moderated by comorbid diseases. In other words, the variation in ToM performance might be modulated by comorbidities. In this context, it has to be emphasized that the ADHD patients in this study had to be free of any comorbidities. This potentially explains the conflicting results of others who did not exclude participants with comorbidities. We assume that other neurodevelopmental, addictive, or affective disorders may lead to limited cognitive perspective taking. The review by Bora and Pantelis (2016) provided numerous data portraying very heterogeneous positions of clinical studies toward the exclusion of comorbid diseases and the extent of treatment and medication. This approach may cause inconclusive results regarding ToM deficits.

Future studies on ADHD should also consider different clinical subtypes of ADHD and their specific (social) cognitive performance. Furthermore, a differentiation in terms of sex would be conceivable, as well as a critical examination of the transitional stages not only from child to adulthood but also from adulthood to the senium.

As Gonzalez‐Gadea et al. (2013) and Bora and Pantelis (2016) showed, executive performance and ToM skills may differ greatly among those with ADHD. Only further investigations can clarify whether ToM deficits or empathy impairments can be mainly explained by executive dysfunction. Furthermore, future research should explicitly address the functional downstream effects of impaired social cognition, that is, what impact do deficits in emotional (empathy) versus cognitive perspective taking (ToM) have on social functioning, at the functional level, and on other outcomes. Future studies should therefore also operationalize the individual's social functioning (e.g., number of unemployment, divorce, law conflicts). This exploration may contribute to a better understanding of causal factors: ToM deficits, empathy impairments, or even ADHD symptomatology itself. Additionally, this might provide relevant information for the development of specific psychotherapeutic treatment strategies. If ADHD symptomatology is held responsible for social deficits, it would make sense to use dialectical‐behavioral treatment approaches. Due to the symptomatic similarity between ADHD and borderline personality disorder, several ADHD therapy manuals include elements such as mindfulness exercises and situational analyses aimed at achieving self‐regulating de‐escalation. Thus, the recurrence of conflict‐laden situations by timely use of socially adequate strategies should be prevented (Philipsen et al., 2010). If social difficulties are based on empathy or ToM impairments, specific training sessions could be used that promote change of perspective (Hofmann et al., 2016).

In addition to cognitive‐behavioral therapy approaches, positive drug effects should be investigated. For example, a study by Maoz et al. (2014) found that ToM and empathy skills improved in children receiving methylphenidate.

The comparatively small sample size of our study bears the risk of being underpowered to show clear differences between ADHD and healthy controls. Therefore, our results require further replication in larger study samples.

## CONFLICT OF INTEREST

BK has received honoraria for lectures from Medice and Servier. In addition, he is an advisory board member for Medice and Servier. MAH has received honoraria from Medice for the compilation of educational brochures. The other authors have nothing to report or to disclose.

## Data Availability

The data that support the findings of this study are available on request from the corresponding author. The data are not publicly available due to privacy or ethical restrictions.
